# A mantidfly in Cretaceous Spanish amber provides insights into the evolution of integumentary specialisations on the raptorial foreleg

**DOI:** 10.1038/s41598-019-49398-1

**Published:** 2019-09-13

**Authors:** Ricardo Pérez-de la Fuente, Enrique Peñalver

**Affiliations:** 1 Oxford University Museum of Natural History, Parks Road, Oxford, OX1 3PW UK; 2 Instituto Geológico y Minero de España (Museo Geominero), C/Cirilo Amorós 42 46004, Valencia, Spain

**Keywords:** Palaeontology, Entomology

## Abstract

Multiple predatory insect lineages have developed a raptorial lifestyle by which they strike and hold prey using modified forelegs armed with spine-like structures and other integumentary specialisations. However, how structures enabling the raptorial function evolved in insects remains largely hypothetical or inferred through phylogeny due to the rarity of meaningful fossils. This is particularly true for mantidflies (Neuroptera: Mantispidae), which have a scarce fossil record mostly based on rock compressions, namely isolated wings. Here, *Aragomantispa lacerata* gen. et sp. nov. is described from ca. 105-million-year-old San Just amber (Spain), representing the oldest and one of the few mantidflies hitherto described from amber. The fossil shows exquisitely preserved forefemoral spine-like structures composed of integumentary processes each bearing a modified seta, and prostrate setae on foretibiae and foretarsi. The fine morphology of these structures was unknown in fossil mantidflies. An assessment of integumentary specialisations from raptorial forelegs across mantispoid lacewings is provided. The present finding reveals how the specialised foreleg armature associated to the raptorial lifestyle in extant mantidflies was present yet not fully established by the Early Cretaceous, at least in some lineages, and provides palaeontological evidence supporting certain evolutionary patterns of acquisition of integumentary specialisations related to the raptorial function in the group.

## Introduction

Among predatory insects, various lineages swiftly strike and hold prey using modified forelegs armed with diverse specialised structures derived from the leg integument, such as spine-like bristles (setae). In extant insects, these specialised, raptorial forelegs are most famously distinctive of praying mantises^1^, but are also present in some aquatic hemipterans^2^, some assassin bugs^3^, some true flies^4–6^, a few beetles^7^, and mantidflies. The latter, also known as mantis lacewings or mantispids (Neuroptera: Mantispidae), are a small insect group with about 400 described extant species distributed in four subfamilies^8,9^. While Mantispinae encompass most of the mantidfly species and are distributed in temperate and intertropical regions from all continents, Calomantispinae and Drepanicinae have a disjunct distribution in the Asia-Pacific region and the New World, and Symphrasinae are only found in the latter^8,10,11^. Mantidfly larvae have straight piercing-sucking mandibulo-maxillary stylets used to absorb the liquefied tissues of their prey and are obligate predators (some even acting as ectoparasites) of spiders and insects, becoming sedentary in late instars^12,13^. Apart from their raptorial forelegs, the most distinctive feature of adult mantidflies ‒also convergent with praying mantises‒ is an elongate prothorax. The mantidfly forelegs are inserted very anteriorly, however, adjacent to the head. It is well known that other lineages of mantispoid neuropterans possess raptorial forelegs, i.e., the Rhachiberothidae (often classified within Berothidae), and two exclusively Cretaceous lineages, the Paraberothinae (considered here a subfamily within Rhachiberothidae) and the Dipteromantispidae^14–18^. Raptorial forelegs are known to be present in other extinct neuropteran groups, i.e., the Mesithoninae (currently within Berothidae)^18,19^ and the Mesochrysopidae^20^.

Although 21 fossils species of adult mantidflies had been hitherto described^21,22^, only four of these are amber inclusions and, from them, just one is Cretaceous^23–25^. *Micromantispa cristata* (from mid Cretaceous Burmese amber) was originally described as a mantidfly^26^ but it can be more comfortably regarded as a paraberothine^27^. On the contrary, fossil mantidflies are much more common as compressions, with 16 species described. Most of them are fully preserved bodies of the extinct Mesomantispinae, known from the Middle‒Late Jurassic to Early Cretaceous of China, Kazakhstan, and Russia^18,22,28–31^, and likely representing a paraphyletic assemblage^31^. Other fossil species assigned to Mantispidae described from compressions correspond to wings, mostly isolated, i.e., three Mesozoic species affiliated to Drepanicinae^32–34^, one Eocene species assigned to Symphrasinae^29^, and three Oligocene species classified in Mantispinae^35–37^. The fossil record of crown thorny lacewings (Rhachiberothinae) is represented by two species from Eocene ambers^38,39^. On the other hand, the fossil record preserved in amber of extinct groups of mantispoids with raptorial forelegs is actually more abundant that that of extant groups. Indeed, most of the amber mantispoids with raptorial forelegs have been assigned to the Paraberothinae (herein considered within Rhachiberothidae), a Cretaceous group currently containing a total of 14 species from Lebanese, Burmese, French (Charente and Bezonnais), New Jersey, Japanese, and Canadian ambers^16,19,40–47^. The affiliation of *Oisea celinea* is uncertain and rests somewhere between Rhachiberothinae and Paraberothinae^16,39,43^. Lastly, the fossil family Dipteromantispidae has six described species from Burmese and New Jersey ambers and one as a compression from the Yixian Fm.^17,42,48,49^.

Here we present a fossil mantidfly with exquisitely preserved integumentary specialisations on the raptorial forelegs, including prostrate setae and spine-like structures composed of integumentary processes each bearing a modified seta. This is the first time that the fine structure of these specialisations has been characterised in extinct mantidflies. The fossil is preserved in Early Cretaceous (middle‒upper Albian, ca. 105 Ma) Spanish amber^50,51^, and represents the oldest and one of the few mantidflies described from amber to date. Although some evolutionary trends of integumentary specialisations related to the raptorial lifestyle in insects have been inferred phylogenetically^1,2,52^, the fossil record able to contribute to the subject is scarce and was hitherto restricted to praying mantises^1,53^.

## Results

### Systematic palaeontology

Order Neuroptera Linnaeus, 1758

Family Mantispidae Leach, 1815

Subfamily Drepanicinae Enderlein, 1910

### Genus *Aragomantispa* gen. nov

LSID, urn:lsid:zoobank.org:act:C12BCD59-8E2C-4C86-AF29-4D19BA9F4E7B.

#### Type species

*Aragomantispa lacerata* sp. nov.

#### Diagnosis

Scape moderately elongate, about 4× longer than wide basally. Forecoxa not particularly elongate, shorter than forefemur (ratio forecoxae/forefemoral length 0.6). Forefemur not laterally flattened ventrally, widening distally and reaching its maximum width slightly beyond its midlength. Forefemur slightly longer than combined length of foretibia and foretarsus (about 1.1×). Forefemur with three types of spine-like structures composed of integumentary processes (IPs) each bearing a modified seta distally, arranged in two longitudinal rows: (1) two ectal and two ental major IPs bearing modified setae; ratio IP length/modified seta length of largest IP (basalmost, ental) 7:1, same ratio of three remaining IPs 3:1; (2) about ten ectal and five ental minor IPs bearing needle-like setae placed on proximal three quarters, ratio IP length/needle-like seta length about 1:3; (3) three ectal and three ental thick, minor IPs bearing thick setae on distal quarter, ratio IP length/thick seta length 3:2. Foretibia slightly arched ventrad, ventrally bearing a single row of closely-spaced prostrate setae, visible only on the distal half of the tibia. Foretarsus pentamerous, with tarsomeres cylindrical and compact. Foretarsomere 1 not particularly elongate, not produced apically. Foretarsomere 5 the longest. Foretarsomeres 1‒4 ventrally with one or two transverse pairs of prostrate setae each. Foretarsal prostrate setae distinct from those on foretibiae, i.e., thicker, with basal stretch erect at about 45° angle and a distal stretch abruptly inclined forwards and running parallel to the tarsus (not directed towards the cuticle). Claws paired and simple (not bifid or multipronged) in all legs. Arolium present in all legs. Meso- and metathoracic legs with tarsomeres 3 and 4 subequal in length. Hind wing with single trichosors along all anterior margin, with costal space very narrow; Sc meeting RA at about 2/3 of the wing length, pterostigmal area hyaline; two ra-rp crossveins before the pterostigmal area, 1rp-ma crossvein straight (not sigmoidal).

#### Etymology

After “Aragón”, name of the Autonomous Community in Spain where the San Just outcrop is located, and *Mantispa*, type genus of Mantispidae. Gender: feminine.

### *Aragomantispa lacerata* sp. nov.

LSID, urn:lsid:zoobank.org:act:B11B14A7-620A-4B6F-A19C-89F29DCADC7B.

Figs 1–5

#### Type material

**Figure 1:**
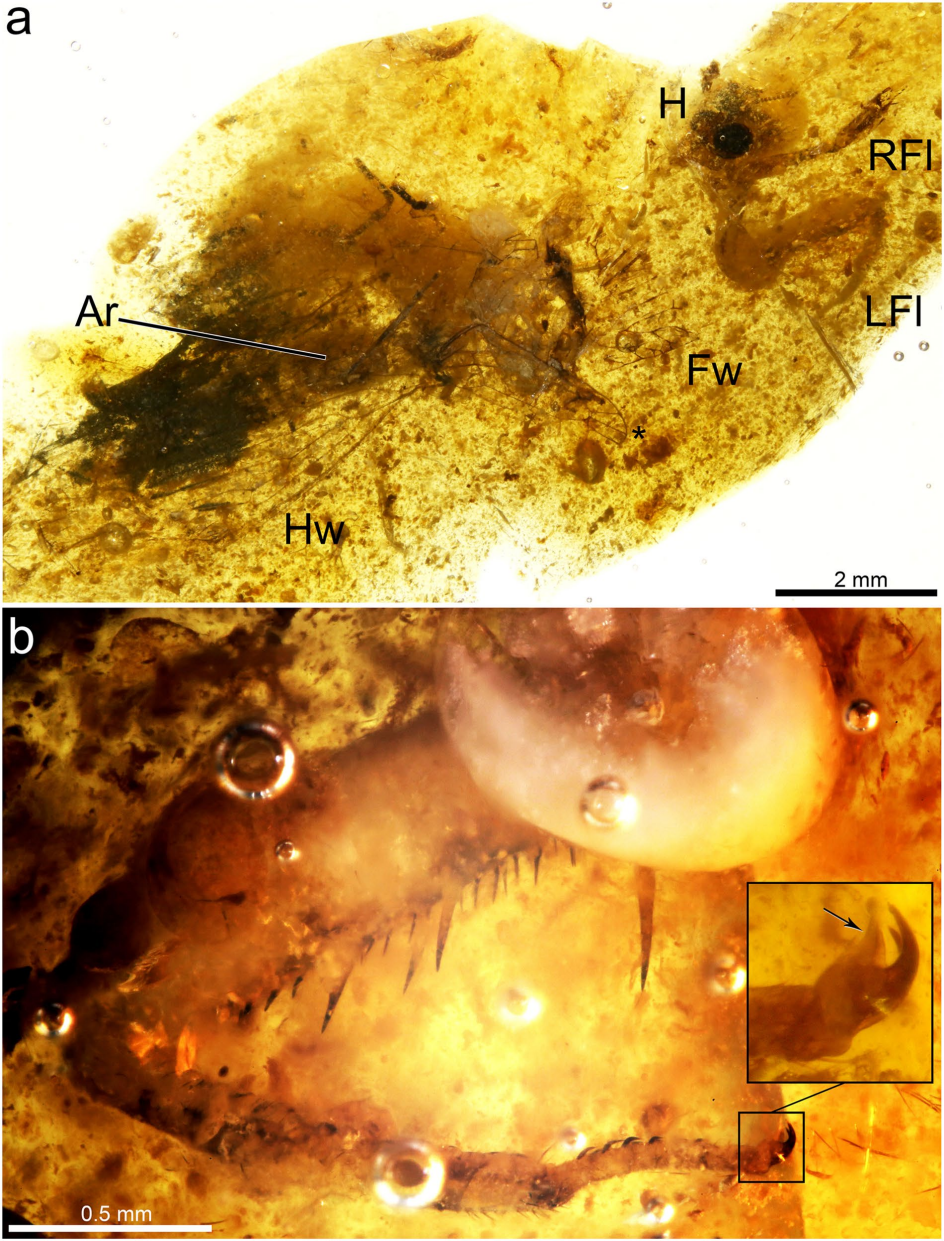
Photomicrographs of *Aragomantispa lacerata* gen. et sp. nov. (Mantispidae: Drepanicinae), holotype SJ-10-22, from San Just amber. (**a**) Dorsolateral habitus, with discernible body parts tagged. (**b**) Left foreleg in lateral (ectal) view, with inset showing pretarsal claws and arolium (arrow). Abbreviations: Ar ‒ Abdominal remains, Fw ‒ Forewing, H ‒ head, Hw ‒ Hind wing, LFl ‒ Left foreleg, RFl ‒ Right foreleg. The asterisk marks a partially preserved snakefly wing (Raphidioptera).

SJ-10-22 from San Just amber. Specimen deposited at the Fundación Conjunto Paleontológico de Teruel-Dinópolis (CPT), Teruel, Spain. Fragmentary specimen preserved in a turbid amber piece prepared in an Epoxy prism measuring 24 × 17 × 2 mm. Although the specimen’s body is badly preserved, the head (excluding the distal antennae), the anteriormost prothorax (including head-prothorax and prothorax-forecoxal articulations), and the forelegs (excluding the right foreleg beyond the mid femur) are preserved in good condition. A basal third portion of a forewing, most part of a hind wing, and three distal fragments of mid-/hind legs are also preserved. Syninclusions: a partial snakefly (Raphidioptera) wing (asterisk in Fig. 1a) and possibly fragments of its legs (one of them showing distinct maculations); a small hymenopteran, and a few other indeterminate insect fragments (legs, eyes, microlepidopteran scales, etc).

#### Diagnosis

As for the genus (see above).

#### Description

Sex unknown. Body medium-sized, inferred length 8‒9 mm from mandibles to end of the abdomen (Fig. 1a).

**Figure 2:**
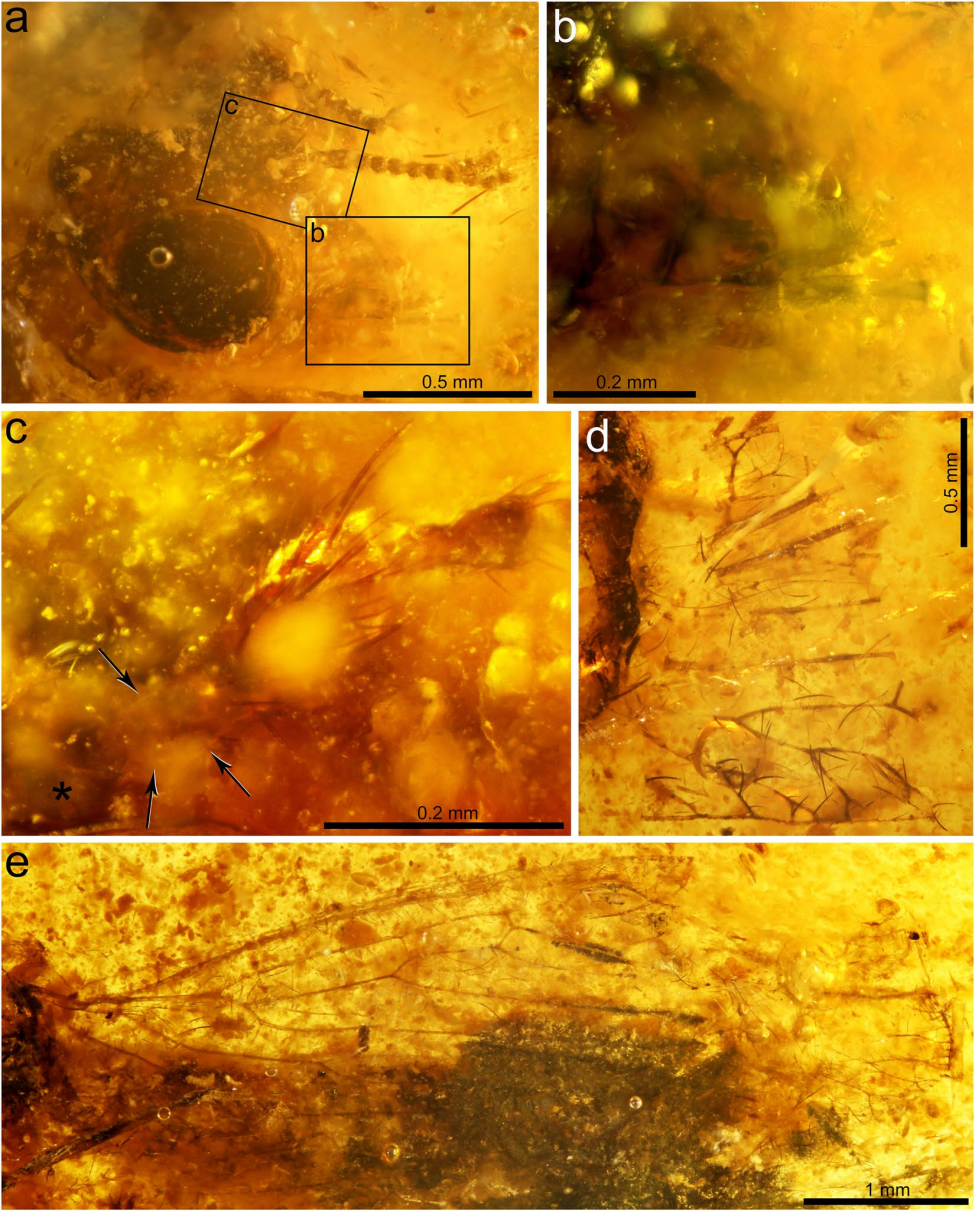
Photomicrographs of *Aragomantispa lacerata* gen. et sp. nov. (Mantispidae: Drepanicinae), holotype SJ-10-22. (**a**) Head in dorsolateral view. Areas depicted in subfigures (**b**) and (**c**) have been framed. (**b**) Detail of mouthparts. (**c**) Detail of base of the right antenna, showing antennal insertion (asterisk), scape (its base delimited by arrows), and pedicel. (**d**) Preserved forewing fragment. (**e**) Hind wing.

**Figure 3:**
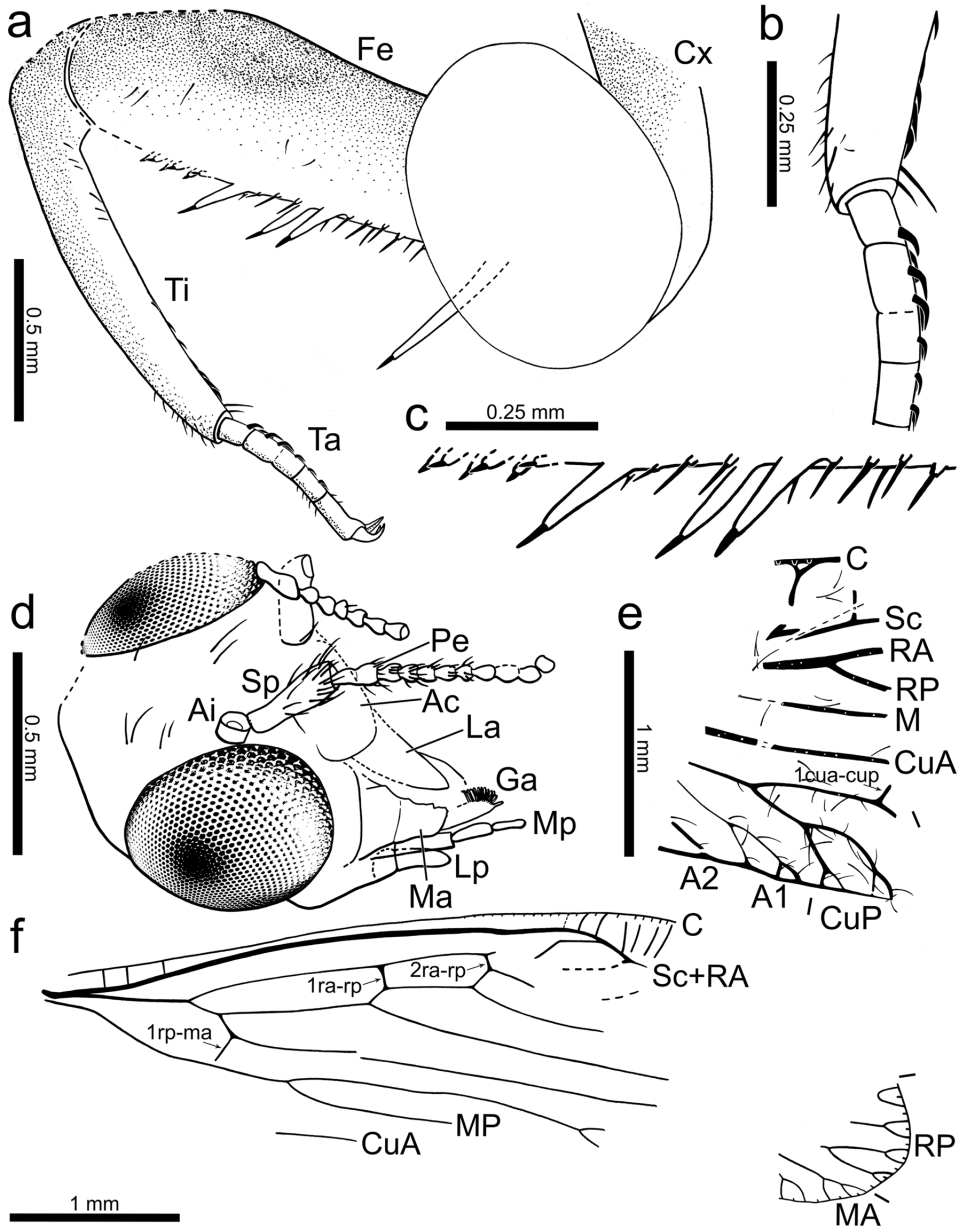
Camera lucida drawings of *Aragomantispa lacerata* gen. et sp. nov. (Mantispidae: Drepanicinae), holotype SJ-10-22. (**a**) Right foreleg in lateral (ectal) view. (**b**) Detail of prostrate setae visible on right foretibia (top) and foretarsus (bottom). (**c**) Detail of spine-like structures (different types of integumentary processes each bearing modified setae) on right forefemur. Proximal is on the right side. (**d**) Head in dorsolateral view. (**e**) Preserved forewing fragment. (**f**) Hind wing. Abbreviations: A1, 2 – anal veins, Ac – anteclypeus, Ai – antennal insertion, C – costal vein, CuA – cubital anterior vein, CuP – cubital posterior vein, Cx – (fore)coxa, Fe – (fore)femur, Ga ‒ galea, La ‒ labrum, Lp ‒ labial palp, M – media vein, Ma – Mandible, Mp ‒ maxillary palp, Pe ‒ Pedicel, RA – radial anterior vein, RP – radial posterior vein, Sc – subcostal vein, Sp ‒ scape, Ta – (fore)tarsus, Ti – (fore)tibia.

Head transverse, width not measurable. Vertex only slightly raised, not distinctly domed, lacking tubercles, sculpturing not discernible, with sparse setae as preserved. Post-ocular margin narrow. Eyes large, ovoid, bulging, 0.55 mm long, 0.41 mm high (Figs 2a and 3d). Mandibles relatively small, 0.24 mm long. Galea with distal digitation distinct, bearing hooked setae. Last maxillary palpomere shorter than the preceding palpomere (Fig. 2b). Antennae incomplete, with at least 13 flagellomeres; antennal insertion prominent, subcontiguous to the anterior eye margin. Scape moderately elongate, ca. 4× longer than wide (basal width), i.e., slightly more than twice the pedicel length and the length of about five flagellomeres, distally expanding, 0.30 mm long, 0.08 mm wide basally, 0.11 mm wide distally; with a whorl of setae distally, setae particularly elongate dorsally (Figs 2c and 3d). Pedicel elongate, 0.13 mm long; basal half cylindrical, 0.04 mm wide, distal half expanding, maximum width 0.06 mm; preserved flagellomeres subquadrate, slightly goblet-shaped, 0.05 mm long, 0.05 mm wide (Figs 2c and 3d).

**Figure 4:**
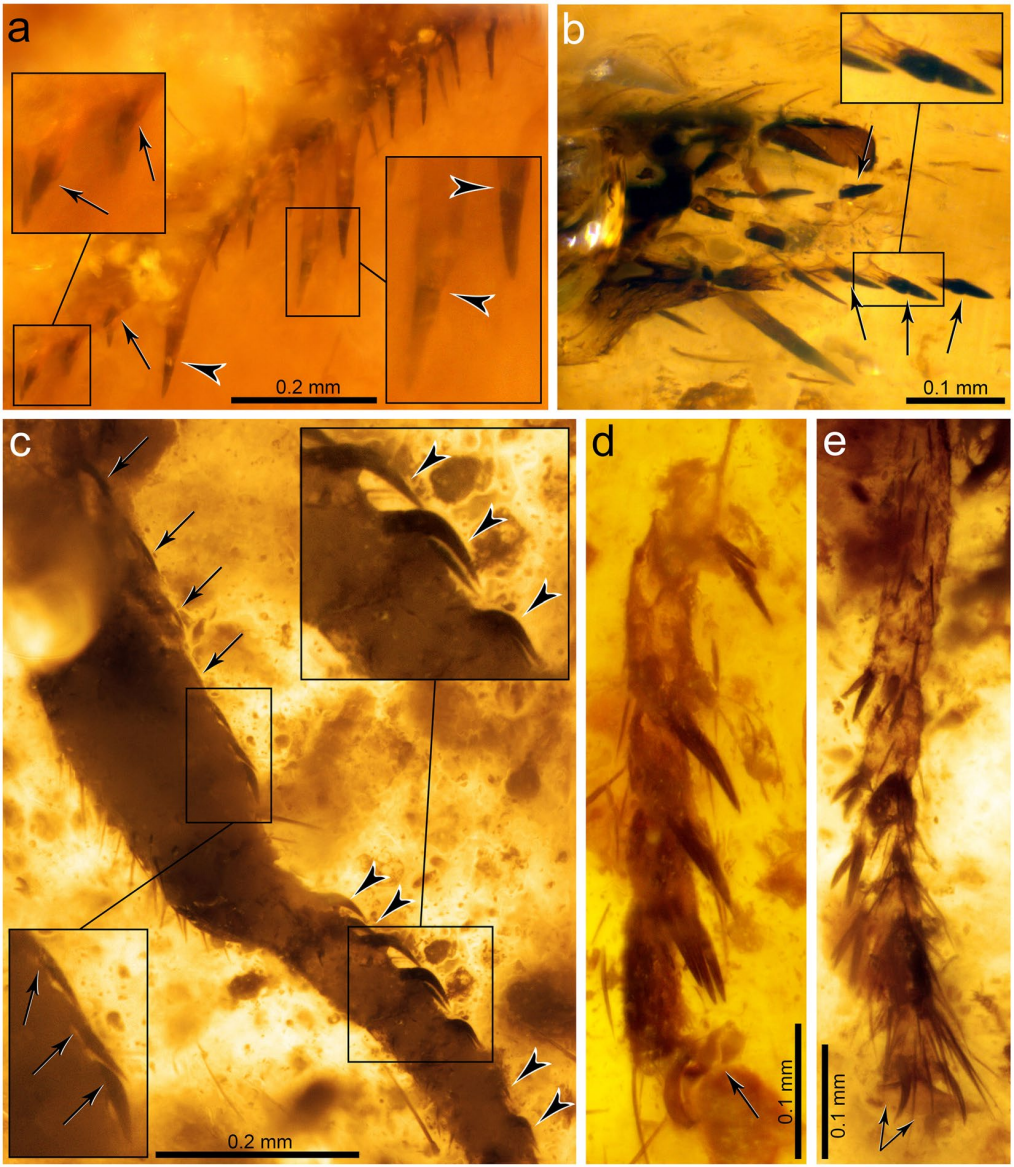
Integumentary specialisations from the legs of *Aragomantispa lacerata* gen. et sp. nov. (Mantispidae: Drepanicinae), holotype SJ-10-22. (**a**) Spine-like structures composed of integumentary processes (IP) each bearing a modified seta on the right forefemur, imaged in lateral (ectal) view. Proximal is on the upper right corner. Left inset shows the two rows of minor IPs bearing thick setae (not fully visible) placed at the forefemur’s distal quarter (arrows point at the insertions of two thick setae on their respective IPs), whereas right inset expands the distal end of two of the major IPs bearing modified setae with a 3:1 length ratio between the two components (arrowheads mark the insertion of the modified setae). (**b**) Integumentary specialisations on the left forefemur imaged in dorsal view (uppermost cuticle not preserved). Inset shows a minor IP bearing a thick seta with a 3:2 length ratio between the two components. (**c**) Prostrate setae on the right foretibia (bottom left inset, arrows) and foretarsus (top right inset, arrowheads). (**d**) Isolated mid- or hind tarsus, showing enlarged setae on plantar surfaces. Arrow points to arolium. (**e**) Tarsus from isolated mid- or hind leg, showing enlarged setae on plantar surfaces. Arrows point to arolium.

Thorax mostly not preserved. Only distal prothorax preserved, in connection to the head. Prothoracic legs raptorial, attached to the anteriormost portion of the thorax (Figs 1b and 3a). Forecoxa not particularly elongate, 1.23 mm long, shorter than forefemur, ratio forecoxa/forefemur length = 0.6, without an evident transverse sulcus. Foretrochanter 0.32 mm long. Forefemur not flattened ventrolaterally, varying in thickness throughout, i.e., dorso-ectally projecting until reaching maximum thickness (approximately 0.38 mm) beyond its midlength. Forefemur 2.05 mm long, about 1.1× the combined length of foretibia and foretarsus (1.82 mm). Forefemur ventrally armed with three types of spine-like structures composed of IPs each bearing a modified seta, arranged in two longitudinal rows, all slightly directed forwards (Figs 1b, 3a,c and 4a,b): (1) two ectal and two ental major IPs each bearing a modified seta, basalmost of these structures the largest, more than twice as long as remaining major IPs bearing modified setae (0.53 vs 0.24 mm long), inserted on proximal quarter of the femur length; ratio IP length/modified seta length of basalmost IP 7:1, same ratio of three remaining IPs 3:1; modified seta about 3.5–4× longer than wide basally (emerged portion of seta); (2) about ten ectal and five ental minor IPs bearing needle-like setae placed on proximal three quarters; ratio IP length/needle-like seta length about 1:3; needle-like setae 0.10 mm long, 6–6.5× longer than wide basally (emerged portion of seta); (3) three ectal and three ental minor IPs bearing thick setae placed on distal quarter; ratio IP length/thick seta length 3:2; thick setae 0.08 mm long, 2× longer than wide basally (emerged portion of seta). Foretibia slightly arched ventrad, 1.20 mm long, 0.18 mm high. Foretibia ventrally with closely-spaced prostrate setae (adpressed and facing forwards) arranged in a single row, visible only on the distal half of the tibia as preserved (Figs 1a, 3a,b and 4c). Foretibia lacking spine-like setae or IPs. Foretarsus pentamerous, 0.62 mm long, 0.06 mm wide. Foretarsomeres cylindrical, compact. Foretarsomere 1 not particularly elongate, not produced apically, not bearing dorsal spine-like setae. Foretarsomere 5 the longest. Foretarsomere 1 0.12 mm long, 2 0.11 mm, 3 0.07 mm, 4 0.11 mm, and 5 0.13 mm. Foretarsomeres 1‒4 ventrally with one (foretarsomere 1 and 3) or two (foretarsomere 2 and 4) transverse pairs of prostrate setae each, lacking spine-like setae. Foretarsal prostrate setae distinct from those on foretibia, thicker, distally raised (not adpressed to the tarsal cuticle), i.e., with basal stretch erect at about 45° angle and distal stretch abruptly inclined forwards and running parallel to the tarsus (not directed towards the cuticle), 0.07 mm long (Figs 3a,b and 4c). Prothoracic leg with two pretarsal claws, simple (not bifid), sturdy, 0.08 mm long, 0.07 mm high, with relatively large arolium present between them (Fig. 1b), slightly surpassing the tip of the claws. Meso- and metathoracic legs fragmentarily preserved. Mid-/hind tarsomere 2 longer than wide. Mid-/hind tarsomere 3 as long as tarsomere 4, both subquadrate (not elongate). Mid-/hind tarsomeres with combs of six to eight enlarged setae on distal plantar surfaces (Fig. 4d,e), particularly strong in one of the preserved tarsi; two pretarsal claws present, simple (not bifid), with an arolium between them, 0.07 mm wide. Metathoracic leg (left?) with femur 1.28 mm long; tibia without spurs, 1.90 mm long.

Forewing with only a fragment from its basal third preserved, 1.60 mm wide, with weakly marked trichosors likely present anteriorly (Figs 2d and 3e). Costal space with two preserved crossveins, the basalmost narrowly forked. Crossvein 1cua-cup preserved, situated beyond branching of RP. Posterior CuP branches shallowly twigged at wing margin. Vein 1A with one simple branching, its anterior branch shallowly twigged.

Hind wing 5.21 mm long, about 1.55 mm wide, with single trichosors between veins along its preserved margin (Figs 2e and 3f). Apex rounded. Costal space very narrow, with a few costal veinlets preserved proximally and around the pterostigmal area, all simple (not twigged). Costal veinlets not apparent. Pterostigmal area hyaline. Sc meeting RA at about 2/3 of the wing length. Two ra-rp crossveins before Sc reaches RA. RP with, at least, four branches, without gradate series of crossveins visible. Crossvein between stems of RA and MA absent, the basalmost crossvein present (1rp-ma, also known as “basal piece of MA” or “b vein” in older nomenclatures) located almost immediately after the branching of the basal fork of RP, straight (not sinuous). Branching of MA and MP distad the basalmost branching of RP. Cubital and anal veins not preserved.

Abdomen barely preserved, ca. 4.20 mm long. Genitalia not preserved.

#### Age and locality

San Just amber, northeastern Spain (Teruel Province)^50^. Dated as middle‒upper Albian^51^, but most likely upper Albian according to new extensive, unpublished data on palynomorphs.

#### Etymology

Specific name is after Latin verb *lacerare*, meaning “to tear to pieces, to shatter, to destroy”, in its feminine, singular participle perfect passive conjugation, referring to the fragmentary and disintegrated appearance of the holotype’s body.

## Remarks

**Figure 5:**
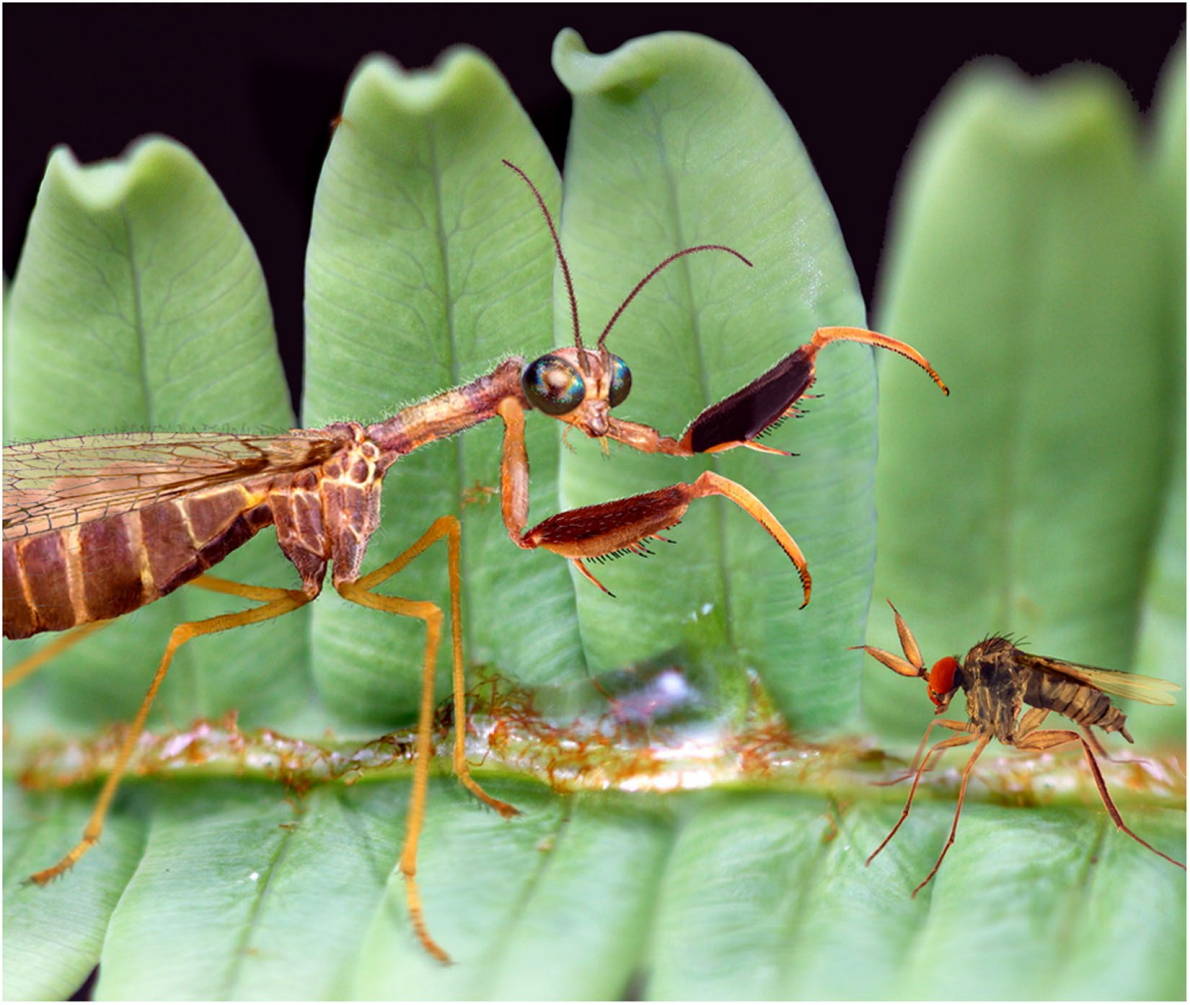
Reconstruction of *Aragomantispa lacerata* gen. et sp. nov. (Neuroptera: Mantispidae) striking a potential prey, an *Alavesia* sp. fly, on a hypothetical gleicheniacean fern. Antennal length, thoracic (including pronotal shape and the proportions of meso- and metathoracic legs) and abdominal morphology, striking pose and colouration of the new taxon based on extant mantidfly relatives. Species classified within the genus *Alavesia* have been found in two Spanish amber localities^74^, and the fern group is recorded as trichome inclusions and spores within the sediments associated to Spanish amber^75^; both were most likely abundant in the Iberian amber forest.

**Figure 6:**
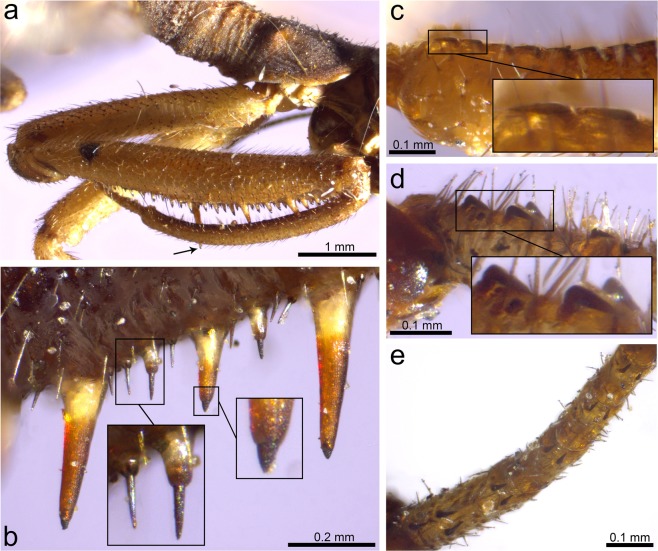
Specialised integumentary structures on the prothoracic leg of *Theristria delicatula* (Mantispidae: Drepanicinae; extant). (**a**) Lateral (ectal) view of the right prothoracic leg (specimen 1). Arrow points to the tip of the major IP. (**b**) Lateral view of the distal half of a femur showing diversity of spine-like integumentary specialisations (specimen 2), including three major IPs each bearing a partly invaginated chitinous cone (Stitz organ) (right inset), minor IPs bearing modified setae with length ratio between the two of ca. 1:1 (left inset, right), and spine-like setae inserted on a subglobular base (left inset, left). (**c**) Lateral view of the ventrodistal part of the tibia, showing row of prostrate setae, with inset on two distalmost prostrate setae (specimen 1). (**d**) Lateral view of ventral side of foretarsomeres 5 (left) and 4 (right), with inset showing the basalmost prostrate setae (specimen 2). (**e**) Ventral view of a foretarsus (specimen 2). All images are ^©^Oxford University Museum of Natural History, released under a CC BY license.

**Figure 7:**
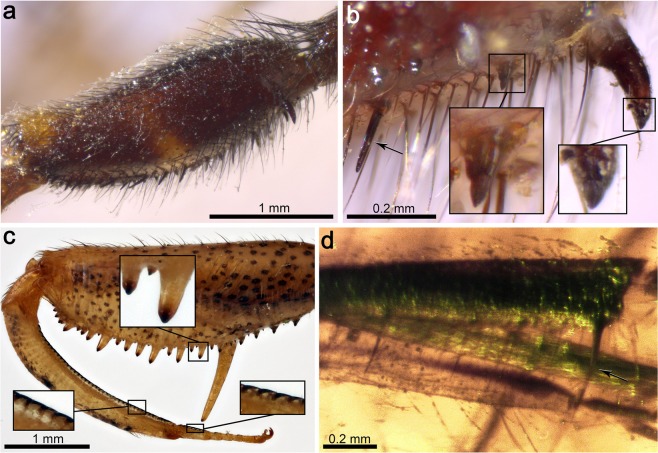
Specialised integumentary structures on the prothoracic legs of extant and extinct mantispoids. (**a**) *Anchieta notha* (Mantispidae: Symphrasinae; extant), lateral view of forefemur. (**b**) Same specimen, detail of the basal part of forefemur in lateral view, showing a spine-like seta with a globulose base of insertion (arrow) and two IPs, i.e., a small conical process (left inset) and the basalmost (major) process (right inset), each bearing a chitinous cone (Stitz organ). (**c**) *Nolima victor*, syntype (Mantispidae: Calomantispinae; extant). Lateral view of forefemur, foretibia, and foretarsus, with an inset of minor forefemoral IPs bearing chitinous cones (Stitz organs) (top inset) and row of prostrate setae on foretibia (bottom left inset) and foretarsus (bottom right inset). (**d**) *Eorhachiberotha burmitica*, holotype (Rhachiberothidae: Paraberothinae; extinct). Lateral view of the forefemora and foretibia. Arrow points at the insertion of a modified seta on the basalmost forefemoral IP. Images (**a**) and (**b**) are ^©^Oxford University Museum of Natural History, (**c**) and (**d**) are ^©^The Trustees of the Natural History Museum, London; all images are released under a CC BY license.

The latest phylogenetic assessment of Mantispidae^11^ established four apomorphic characters for the family: (1) pronotum elongate posterior to forecoxae^54^ (Figs 5 and 6a); (2) pronotum tubular (ventrally fused)^54^ (Fig. 6a); (3) foretibiae with prostrate setae^10,55^ (lost in Mantispinae) (Figs 6c and 7c); and (4) meso- and metathoracic legs with third and fourth tarsomeres subequal in length^54^. The presence of prostrate setae on the foretibia (and the foretarsus) (Figs 3b and 4c) firmly accommodates the new fossil species within Mantispidae. The third and fourth tarsomeres of the meso-/metathoracic legs are subequal in length (Fig. 4d,e), although this character is rather subtle to assess and has not been evaluated in most fossil mantispoids with raptorial forelegs such as in paraberothines. The presence of a tubular pronotum and its elongation posterior to the forecoxae insertion cannot be assessed in the new species due to preservation. The absence of spine-like setae on the ventral side of the foretibia (and the foretarsus) further discards a relationship of the new fossil with paraberothines^19^, which lack prostrate setae (see discussion).

Multiple characters present in *Aragomantispa lacerata* gen. et sp. nov. rule out its affiliation to the mantispid subfamilies Symphrasinae, Calomantispinae, and Mantispinae, and suggest accommodation within the remaining mantispid subfamily, the Drepanicinae (although see below)^10,11^. These characters are: (1) absence of forefemur laterally compressed ventrally (presence is synapomorphic of Calomantispinae and Mantispinae), (2) combined length of foretibia and foretarsus about as long as that of forefemur (clearly shorter in Mantispinae), (3) pentamerous foretarsi (tetramerous foretarsi are apomorphic for Symphrasinae), (4) foretarsomere 1 not distally produced (distally produced in a claw-like process present, likely as an apomorphy, in Symphrasinae), (5) two pretarsal claws in all legs (one pretarsal claw on the prothoracic leg is apomorphic in Mantispinae)^11^, and (6) arolia present in all legs (absent in the prothoracic leg of Mantispinae). Nevertheless, the character states noted above are considered plesiomorphic for Drepanicinae, the monophily of which is supported by genitalic characters^11^, which are not assessable in the new fossil due to its fragmented condition.

**Figure 8:**
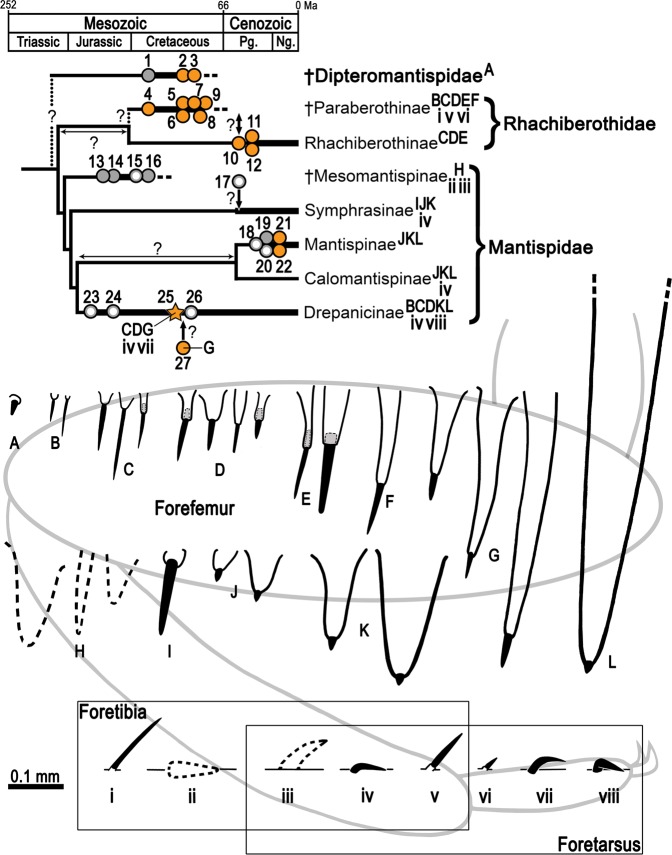
Fossil record of mantispoid neuropterans with raptorial forelegs (excluding Mesithoninae) and types of integumentary specialisations present on the forefemora (A‒L), foretibiae and/or foretarsi (i‒viii) for each of the groups, including extinct and extant taxa unless fossils are directly tagged (only in Drepanicinae). Rock fossils appear in grey (isolated wings depicted as empty circles) and amber inclusions in orange, including *Aragomantispa lacerata* gen. et sp. nov. (star, Spanish amber). Phylogenetic relationships based on Liu *et al*.^1^. Divergence times are conjectural but consistent with those obtained for Rhachiberothidae and Mantispidae^71,76^. Phylogenetic, temporal, and taxa assignation uncertainty is marked with question marks on arrows or discontinuous line. Tibial spurs from extant Rhachiberothidae and foretibial “peg-like protrusions” of *Doratomantispa* have been excluded. Fossil occurrences (numbers 1 to 27) and taxa from which depicted types of integumentary specialisations have been extracted are provided in the Supplementary Text. Forefemoral integumentary specialisations: A, short and thick spine-like seta; B, spine-like setae; C–G, integumentary processes (IPs) each bearing a modified seta: C minor size (<0.2 mm), ratio IP length/modified seta length 1:2 to 1:5; D, minor size, ratio around 1:1 (ratios of 2:3 and 3:2 also present); E, major size (>0.2 mm), ratio ca. 1:1; F, major size, ratio 2:1; G, major size, ratio 3:1 to 7:1 (basalmost IP); H, mesomantispine spine-like structures (fine structure unknown due to fossilisation as compressions); **I**, large and thick spine-like seta; J‒L, increasingly larger IPs bearing minute chitinous cones (Stitz organs). Foretibial and/or foretarsal integumentary specialisations: i,v,vi, erect spine-like setae; ii,iii, mesomantispine prostrate seta and erect spine-like structure, respectively (fine structure unknown due to fossilisation as compressions); iv,vii,viii, prostrate setae (adpressed to the cuticle for all their length, distal stretch raised and running above the cuticle, or adpressed only at the tip, respectively). All integumentary specialisations at the same scale. Abbreviations: Pg ‒ Palaeogene, Ng ‒ Neogene.

Three characteristics based on the arrangement and microstructure of the integumentary specialisations from the raptorial forelegs support the assignation of the new fossil to the Drepanicinae: (a) a combination of major IPs each bearing a modified seta, the basalmost being the largest and a few of such structures, yet smaller, more anteriorly placed, and numerous minor IPs each bearing a modified seta^55^ (Figs 1b, 3a,c and 4a,b), (b) tarsal prostrate setae arranged in transverse pairs (not forming a single row as in Symphrasinae and Calomantispinae), and (c) foretibial and foretarsal prostrate setae having a different shape, with the latter being thicker, more raised and angulated (Figs 3b and 4c), a state detected in *Theristria delicatula* (Fig. 6d; see types vii, viii in Fig. 8; see discussion) (foretibial and foretarsal prostrate setae shape subequal in Symphrasinae, Calomantispinae, and some Drepanicinae). Similarly, the preserved hind wing venation of the new fossil bears resemblance to that of extant drepanicines^10^, rather than to the other mantispid groups^10^, although that is likely a result of its plesiomorphic condition as well.

Some characters differ between *A*. *lacerata* and extant mantispids, including drepanicines. First, the shape of the scape is more elongate in the new species than in extant mantispids, where the scape is 1‒2× longer than wide. This character is known to have a significant variability within the mantispoid groups (i.e., Berothidae, Rhachiberothidae), even among species treated as congeneric (see *Rhachiberotha*^15^), with the greatest elongation degree (scape up to about 10‒12× longer than wide) found in some paraberothines^15,44^. Other antennal characters, remarkably the number of flagellomeres in Mantispidae, show a high degree of intraspecific variation in extant taxa and are, therefore, not informative^10,55^. Second, the shape of the IPs differs from that present in extant mantispids in that instead of bearing minute Stitz organs on their tip, each bears a larger, modified thick seta that is 3.5–4× longer than wide basally (Fig. 4a). And lastly, although the tarsal prostrate setae have a very similar morphology than those present in some *Theristria* species as noted above, their distal stretch remains raised and running parallel above the cuticle instead of being inclined towards it. In any case, and in spite of these differences, the most conservative approach for now is to classify the new species within the subfamily Drepanicinae. Future findings of more complete fossil material related to the new taxon will clarify that stance. Note that whereas trichosors between veins are entirely lacking in most extant Drepanicinae, Calomantispinae, and Mantispinae^11^, they are present in multiple numbers along the anterio-distal wing margin in the drepanicine *Gerstaeckerella* Enderlein^10,54^ and along the whole wing margin in Symphrasinae^56^. The presence of single trichosors between veins along the entire wing margin is shown by the new species and many other fossil mantispids^25,29,31^.

Within the fossil diversity of Mantispidae, *A. lacerata* is most similar to *Doratomantispa burmanica*, described from late Albian-earliest Cenomanian Burmese amber as a drepanicine^25^ but currently considered of unknown familial relationships^11^. Interestingly, both species share the presence of forefemoral IPs bearing modified setae where the latter is about 4× longer than wide basally (Fig. 4a; see type G in Fig. 8). This condition was not originally described but is discernible from the provided photographs^25^. However, among other characters, *Aragomantispa* gen. nov. differs from *Doratomantispa* in the forecoxae not as elongate, four major IPs on the forefemora (vs. six), presence of forefemoral minor IPs bearing modified (needle-like and thick) setae, foretibial prostrate setae (vs. peg-like protrusions), presence of prostrate setae on foretarsomeres 1‒4, foretarsomere 1 among the shortest (vs. the longest), presence of arolium, and two (vs. 1) ra-rp crossveins before the pterostigmal area in the hind wing.

The placement of *Micromantispa cristata*, from Burmese amber and initially considered a mantidfly^26^, has raised controversy^19,27,57^. We agree with Makarkin^19,27^ in that this taxon is more comfortably accommodated within the Paraberothinae, particularly due to the highly diagnostic presence of spine-like setae on the inner foretibia (and foretarsus), absent in mantidflies, and lack of prostrate setae. Note that “prostrate setae” were described from *M. cristata*’s foretibia, but, surprisingly, from its dorsal surface instead of ventrally^26^. Judging from the photographs offered by the authors, their “prostrate” setae appear to represent the insertions of partly detached, regular setae from the dorsal surface of the foretibia. In any case, as Shi *et al*. noted^57^, it is paramount to keep unveiling diversity of fossil mantispoids with raptorial forelegs in order to elucidate their true relationships, including that of *M. cristata*.

## Discussion

In praying mantises, the foreleg’s spines not only act as physical structures to catch, hold, and direct prey deeper into the femorotibial junction, but some of these –the femoral hinged spines– are also mechanoreceptors that elicit the striking reflex and, once the prey is captured, sense its movements and keep the forefemur and the foretibia closed against each other^1,58^. The femora from the raptorial forelegs of extant mantispids are also equipped with a sophisticated, yet different, sensory equipment: modified setae borne by each spine- or tubercle-like integumentary process (IP) shape minute, partly invaginated chitinous cones^55,59,60^ (Figs 6b, 7b,c; see types J‒L in Fig. 8). These structures were named “Stitz organs” and described as mechanoreceptors hypothesised to derive from sensillae trichoidea based on neurohistological observations^60^. Stitz organs are also known to be present on the pronotum from some drepanicine mantidflies^10,11,55^. In extant symphrasine and drepanicine mantidflies, apart from the IPs bearing a Stitz organ (Figs 6b and 7b,c), other integumentary specialisations exist: long and thick spine-like setae (Lambkin’s “long thick black setae”^10^; see Tjeder, 1959: fig. 249^14^), with bases of insertion that are globulose, are present in Symphrasinae (Fig. 7a,b; see type I in Fig. 8), whereas in extant Drepanicinae, although similar yet smaller spine-like setae with globulose bases of insertion can be present^55^, moderately elongate IPs each bearing a modified seta, with a ratio IP length/modified seta length of ca. 1:1, also exist (Fig. 6b; see types B and D in Fig. 8).

Although the fine structure of the anteriorly directed, spine-like structures on the ventral side of the forefemora is relatively well known for extant mantispoids with raptorial forelegs, it has remained largely overlooked in fossils. This is particularly true in Paraberothinae, the disparity of forefemoral spine-like structures of which includes spine-like setae (type B in Fig. 8) and diverse types of IPs each bearing a modified seta. The latter differ in total size, ranging from about 0.05 to 0.3 mm, as well as in the relative length between the IP and the emerged portion of the modified seta that each bear distally. This includes IPs shorter than the modified seta (ratio IP length/modified seta length 1:2 to 1:5; type C in Fig. 8), IPs equal or subequal in length than the modified seta (ratio IP length/modified seta length around 1:1; types D and E in Fig. 8), and IPs longer than the modified seta (ratio IP length/modified seta length 2:1; type F in Fig. 8). In that regard, re-examination of the holotype of *Eorhachiberotha burmitica* shows how, in spite of not having been originally described likely due to bad preservation^43^, the specimen’s forefemora are at least armed with a basal IP bearing a modified seta, with a length ratio between the two of ca. 1:1 (Fig. 7d). By contrast, the diversity of integumentary specialisations in crown Rhachiberothidae appears to be less disparate (types C‒E in Fig. 8)^14,15^. Furthermore, in Dipteromantispidae, short and thick spine-like setae arranged in a few rows have been described from some forms (type A in Fig. 8), whereas in others there appears to be an absence of forefemoral integumentary specialisations^42,48,49^. The fine structure of the forefemoral “spines” of the mesomantispines remains unknown due to their preservation as rock compressions (type H in Fig. 8).

Prostrate setae refer to strong setae adpressed to the cuticle (i.e., not erect, recumbent) and with apices pointing anteriorly^10,14,54,55,61^ (Figs 4c, 6c,d and 7c). These integumentary specialisations play a mechanical role in the raptorial function of mantidflies by creating a hardened edge towards which the spines of the forefemur slide along, creating a “scissor” effect^10^. As mentioned earlier, the presence of prostrate setae forming a closely-spaced lateroventral ridge ventrally on the foretibia is considered an apomorphy of Mantispidae that was lost in Mantispinae^11^; in the latter, a sclerotised and acute longitudinal rim of the foretibial cuticle itself creates the hardened cutting edge instead^10^. Remaining mantispoid groups with raptorial forelegs lack prostrate setae on the foretibia, instead having spine-like setae (often also present on the foretarsi as well) (Paraberothinae)^16,17^ (see types i, v, vi in Fig. 8) or no specialised setae at all in these leg segments (crown Rhachiberothidae and Dipteromantispidae)^14,42,48,49^. Moreover, prostrate setae are additionally present on the foretarsus of all mantispids but Mantispinae, but their location, arrangement and morphology is variable between these groups. In Symphrasinae, foretarsal prostrate setae are restricted to a longitudinal row on foretarsomere 1, which has a claw-like process distodorsally, and the prostrate setae morphology is the same than that present on the foretibia^14,54,56,59,62,63^. In Calomantispinae, foretarsal prostrate setae are present on foretarsomeres 1–4; those in foretarsomere 1 are arranged in a single row, and their morphology is essentially the same than those on the foretibia (although apparently slightly thicker for some species, such as *Nolima victor*) (Fig. 7c). In Drepanicinae, however, foretarsal prostrate setae are arranged on transverse pairs on foretarsomeres 1‒4^10,11,55^ (Fig. 6e), and the prostrate setae morphology can differ between those on foretibia and those on foretarsus (a circumstance not ascertainable from previous accounts^10,55,63^). Indeed, in some *Theristria* species such as *T. delicatula*, prostrate setae on foretarsomeres 1‒4, instead of being thin, gently curving and completely adpressed to the cuticle for all their length, they are thicker and have a basal stretch that is rather erect and a distal stretch abruptly inclined downwards, therefore being directed towards the cuticle (Fig. 6d). This condition resembles that present in *Aragomantispa lacerata* gen. et sp. nov., although in the latter the distal stretch of the foretarsal prostrate setae does not incline downwards so it remains raised running parallel above the cuticle (Fig. 4c). For other examined drepanicine species, however (*T. storeyi*, *Allomantispa mirimaculata*), morphology between foretibial and foretarsal prostrate setae is essentially the same. Moreover, in the Cretaceous mantidfly *Doratomantispa burmanica*, originally considered a drepanicine but the relationships of which were later considered enigmatic^11^, blunt peg-like (not setae-like) protrusions forming a discontinuous ridge on the inner foretibiae were described^25^. Even if these structures probably correspond to modified prostrate setae^11^, their morphology and arrangement significantly differ from those of the prostrate setae from extant mantispids. Lastly, prostrate setae have been described from the foretibiae and foretarsus of some mesomantispines, all preserved as compressions from Asia^18,21,22,31^; as the fine structure and exact arrangement of these integumentary structures is unknown, their homology with those present in the new taxon (Fig. 4c) and extant mantispids (Fig. 6c–e) should remain contentious.

Comparison between integumentary specialisations from the different insect groups with raptorial forelegs provides some interesting insights. In praying mantises (Mantodea), the foreleg armature related to the raptorial function, apart from the foretibial spur, namely consists of modified (spine-like) setae (=spines) variable in number and development (multiple secondary reductions are known), and only a few taxa have spines that are inserted on elevated IPs (=socketed spines)^1^. Some Cretaceous mantises bear spines (allegedly articulated) on mid- and/or hind legs^53^, resembling the condition present in some plesiomorphic lineages of extant mantises such as *Chaeteessa*^1^. The fossil record of praying mantises reinforces the hypothesis that the spines on the mantodean foreleg originated from setae, and that the former are homologous with the setae from the blattodean (cockroach) foreleg^1^. In hemipterans, the raptorial forelegs of aquatic nepomorphan hemipterans (i.e., Belostomatidae, Gelastocoridae, Naucoridae, Nepidae, and Potamocoridae) usually show a lower degree of development of integumentary specialisations^3^. However, those present in assassin bugs (Reduvioidea) are widely diverse, including highly specialised structures such as the “fossula spongiosa” and the use of sticky secretions produced by specialised setae^2^. Even if the disparity of modified spine-like setae and IPs bearing modified setae from assassin bugs is comparable to that identified herein from extinct mantispoids, modified setae do not appear to be as reduced as the minute chitinous cones representing the Stitz organs in extant mantispids^64–67^. In true flies, thickened setae are present in raptorial forelegs of both empidid flies^5^ and predaceous biting midges, the latter also showing moderately elevated IPs in some cases, including in some fossil forms^4,68^. Furthermore, in the genus *Ochthera* (Ephydridae) forefemora have an armature of thickened setae some of which are inserted on moderately elevated IPs; remarkably, *Ochthera* also shows prostrate setae (apparently in a single row) along the foretibial spur^6,69^, a condition highly resembling that present on the spur-like foretarsomere 1 of symphrasine mantispids^56^. Lastly, some staphylinid beetles have raptorial forelegs and show striking behaviour but it is the foretarsus that bears adhesive setae^7^.

Although prostrate setae are one of the few apomorphic characters of mantidflies, their fine structure had not been hitherto described from any fossil representative. *Aragomantispa lacerata* gen. et sp. nov. proves how Cretaceous mantidflies possessed types of prostrate setae comparable to those of their extant counterparts. On the other hand, the most widespread mechanism by which spine-like structures have developed in the forelegs of insects associated to the raptorial function is through the modification, namely enlargement and thickening, of setae. However, a different trend that has contributed to shape functional spines in some insect lineages with raptorial forelegs is the development of integumentary processes each of them elevating the modified setae. Although such structures are nowadays present in some reduviid, mantodean, and dipteran lineages, in no group they are as pervasive as in extant mantidflies, where modified setae reach their minimum size in the form of minute chitinous cones, or Stitz organs. It had been previously hypothesised that the Stitz organs were the result of the reduction of sensillae trichoidea. The findings presented herein provide palaeontological evidence in support of that hypothesis. In that regard, two character states of integumentary specialisations related to the raptorial function in the new species, i.e., (1) foretarsal prostrate setae distally raised and running parallel above the cuticle, and (2) major forefemoral integumentary processes each bearing a modified seta about 4× longer than wide basally (allegedly also present in *Doratomantispa*), could represent transitional stages between the plesiomorphic, more generalised (seta-like) conditions and those present in extant mantidflies, where (1) foretarsal prostrate setae are, at least distally, adpressed to the cuticle and (2) forefemoral integumentary processes bear Stitz organs. In any case, only a phylogenetic analysis including both raptorial and non-raptorial mantispoids once more fossil material is unearthed and studied will be able to ascertain the intermediate nature of these character states. The variability of integumentary specialisations from the ventral surface of the femora, tibiae, and tarsi from the raptorial forelegs of mantispoids showcased herein demonstrates the need to provide detailed descriptions of these structures ‒a practice that has been scarcely done in the past, particularly for fossils‒ in order to account for additional characters of potential phylogenetic significance. Fossils like *A. lacerata* are invaluable for providing data to help elucidate the complex evolutionary history of mantispoids, including that of the traits enabling the raptorial lifestyle.

## Material and Methods

### Taxa examination

The new fossil specimen was isolated within a small amber piece and prepared in Epoxy resin. A Discovery.V12 Zeiss stereomicroscope, and two compound microscopes (an Olympus BX51 and a Zeiss AXIO) were used to examine the specimens. The new fossil specimen was drawn using a camera lucida attached to the stereomicroscope and to the Olympus BX51 compound microscope. Specimens were photographed using an Axiocam 105 colour digital camera attached to both the stereomicroscope and the Zeiss AXIO. Series of images were taken with the software ZenPro v.2.3 and stacked using the software Helicon Focus v.6.8.0. This published work and the associated nomenclatural acts have been registered in ZooBank, the proposed online registration system for the International Code of Zoological Nomenclature. The ZooBank LSIDs (Life Science Identifiers) can be resolved and the associated information viewed through any standard web browser by appending the LSID to the prefix “http://zoobank.org/”. The LSID for this publication is 93EF62A7-75C8-4916-A275-CEA9D22D91A3, and those of the associated nomenclatural acts are C12BCD59-8E2C-4C86-AF29-4D19BA9F4E7B (*Aragomantispa* gen. nov.) and B11B14A7-620A-4B6F-A19C-89F29DCADC7B (*A. lacerata* sp. nov.).

### Other material examined

Fossil material: *Eorhachiberotha burmitica* Engel, 2004, holotype. Tags: “NHM Palaeont. Dept. In. 20177”; “Brit. Mus. Geol. Dept. In. 20177.” “Pres. R.O.J. Swinhoe, 3 July 1920”. Pinned (extant) specimens housed at the Oxford University Museum of Natural History (OUMNH): Subf. Drepanicinae – (**1**) *Theristria delicatula* (Westwood, 1852), specimen 1. Tags: “W. AUSTRALIA. Towranna Plains, betw. Yule R. and Sherlock R. Capt. Jan. – May ’98 by E. Clement, Ph.D. Purchased 1899.”, “Standing over: *Mantispa delicatula* (Westwood) ex. Hope-Westwood colln. Ox. Uni. Mus. Nat. Hist. (OUMNH)”, “1899 3034”; (**2**) *Theristria delicatula* (Westwood, 1852), specimen 2. Tags: “*Theristria delicatula* (Westw.) ♂ Gen.prep. by Ragnar Hall 7.IV.1983”, “Standing over: *Mantispa delicatula* (Westwood) ex. Hope-Westwood colln. Ox. Uni. Mus. Nat. Hist. (OUMNH)”; Subf. Symphrasinae – (**3**) *Anchieta notha* (Erichson, 1839). Tags: “Standing over: *Mantispa notha* (Erichs.) ex. Hope-Westwood colln. Ox. Uni. Mus. Nat. Hist. (OUMNH)”, “Det. R.G. Beard 1968. ♀ abdomen + genitalia. Prepared 20-VI-1968 Beard”; (**4**) *Plega* sp. Tags: “ex. Hope-Westwood colln. Pres.1849–1857. OX. UNI. MUS. NAT. HIST. (OUMNH)”, “Sept.4.1907.W.ARIZONA Prescott. R.E.Kunzé. Pres 1913.”, “1913 1123”; Subf. Mantispinae – (**5**) *Dicromantispa interrupta* (Say, 1825). Tags: “Standing over: *Mantispa interrupta* (Say) ex. Hope-Westwood colln. Ox. Uni. Mus. Nat. Hist. (OUMNH)”. Pinned (extant) specimens housed at the Natural History Museum, London (NHM): Subf. Calomantispinae – (**6**) *Calomantispa spectabilis* Banks, 1913. Tags: “det. Ragnar Hall 1983”, “Brit. Mus. 1939–45”; (**7**) *Nolima praeliator* Navás, 1914, holotype. Tags: “Omilteme, Guerrero, 8000 ft, Aug. H.H. Smith”, “Godman-Salvin Collection 1913–214”; (**8**) *Nolima pugnax* (Navás, 1914), holotype. Tags: “S. Geronimo, Guatemala”, “Godman-Salvin Collection 1913–214”; (**9**) *Nolima victor* Navás, 1914, syntype. Tags: “Xucumanatlan, Guerrero, 7000 ft, July. H.H. Smith”, “Godman-Salvin Collection 1913–214”; Subf. Drepanicinae – (**10**) *Allomantispa mirimaculata* Liu & Ohl, 2015, holotype, ♀; (**11**) *Theristria storeyi* Lambkin, 1986. Tags: “det. R. Hull (HRNS) 1987”, “Australia: N. Queensland, Station Creek via, Mt. Carbine”, “23.XII.1971 A.WALFORD-HUGGINGS”, “B.M. 1972–3314”.

## Nomenclatural and Taxonomic Considerations

General nomenclature for Mantispidae follows that of Lambkin^10^. Nomenclature on wing venation follows the revision by Breitkreuz *et al*.^70^ using wing tracheation. These authors showed that MA does not fuse to the last branch of the RP (or “Rs”, “radial sector”) and, therefore, the so-called “basal piece/stem of MA” or “b vein”, which sometimes can be sinuous (sigmoid) in Neuroptera, is a crossvein^70^. For nomenclature on specialised integumentary structures associated to the raptorial forelegs, Poivre’s works are followed for the Stitz organs of extant mantispids^55,60^. In that regard, the use of the term “spine”, “teeth”, “denticle”, or “spine-like seta”^14,42,55^, among others, are far from being satisfactory. It is necessary to use a more precise terminology able to account for the disparity present in deep time Mantispoidea and identified herein. Thus, it is proposed to use the descriptive term “integumentary process (=IP) bearing a modified seta” for the forefemoral structures comprising a more or less elevated integumentary base (=pedestal, socket) shaping an elongate truncate cone or a rounded tubercle, on the tip of which a thickened and variably elongate seta is inserted (e.g., see Tjeder, 1959: fig. 232^14^ and Poivre’s works^55,60^). The term “spine-like seta” is restricted herein to modified seta inserted on regular (globular) bases, not on elevated IPs (*contra* Makarkin^19^). We refrain to use the term “cuticular spines” used by Lambkin^10^ for the IPs where the modified setae correspond to minute chitinous cones named Stitz organs of extant mantispids as they can be interpreted as structures fully composed of leg cuticle, i.e., not bearing any structure derived from setae.

Although still a matter of contention, herein Rhachiberothidae are considered a separate family^15^ following the latest phylogenetic analyses where they have been recovered as a lineage distinct from Berothidae and Mantispidae^11,71^ (although see Winterton *et al*.^72^). Furthermore, Paraberothinae Nel, Perrichot, Azar & Néraudeau, 2005 is considered a valid taxon following the works of Makarkin and Kupryjanowicz as well as Makarkin’s^19,39^ but within Rhachiberothidae (instead of Berothidae) following Nel *et al*.^16^ and more recent works that have considered the fossil forms classified within Paraberothinae as rhachiberothids^11,73^. Acknowledging that the current paraberothine diversity would appear to represent a grade leading to extant rhachiberothids and pending for a phylogenetic analysis where paraberothines are included, the group is tentatively regarded herein as sister to the Rhachiberothinae (=crown Rhachiberothidae).

## Supplementary information

Supplementary Information
