# Supplementary material for: A mantidfly in Cretaceous Spanish amber provides insights into the evolution of integumentary specialisations on the raptorial foreleg
Source: Sci Rep. 2019 Sep 13;9:13248. doi: 10.1038/s41598-019-49398-1 (PMC6744510; doi:10.1038/s41598-019-49398-1)
Supplement: Supplementary file 1 — Supplementary Information [file 41598_2019_49398_MOESM1_ESM.pdf]

Supplementary Information for

A mantidfly in Cretaceous Spanish amber provides insights into the evolution of integumentary specialisations on the raptorial foreleg

Ricardo Pérez-de la Fuente & Enrique Peñalver

Supplementary Text – Expanded information for Figure 8

A. Fossil occurrences of mantispoids with raptorial forelegs (excluding Mesithoninae): (1) Yixian Fm., China, Barremian – *Dipteromantispa brevisubcosta*[17]; **(2)** Burmese amber, Myanmar, late Albian to earliest Cenomanian – *Burmodipteromantispa jiaxiaoae, Halteriomantispa grimaldii*, and *Mantispidipterella longissima*[48,49]; **(3)** New Jersey amber, USA, Turonian – *Jersimantispa henry* and *Mantispidiptera enigmatica*[42,49]; **(4)** Lebanese amber, Barremian – *Chimerhachiberotha acrasarii, Paraberotha acra, Raptorapax terribilissima*, and *Spinoberotha mickaelacrai*[16,41,44]; **(5)** Archingeay-Les Nouillers and Bezonnais amber, France, latest Albian to early Cenomanian – *Retinoberotha stuermeri* and *Alboberotha petrulevicii*, respectively[16,40]; **(6)** Burmese amber – *Creagroparaberotha groehni, Eorhachiberotha burmitica*, Paraberothinae sp., *Micromantispa cristata*, and *Scoloberotha necatrix*[19,26,27,43,45,57]; **(7)** New Jersey amber – *Rhachibermissa phenax* and *R. splendida*[42,45]; **(8)** Kuji amber, Japan, middle Santonian – *Kujiberotha teruyukii*[47]; **(9)** Canadian amber, Campanian – *Albertoberotha leuckorum*[46]; **(10)** Oise amber, France, earliest Eocene – *Oisea celinea*[16]; **(11)** Amber from the Norfolk coast (presumably Baltic amber in origin), UK, Lutetian – *Whalfera venatrix*[38,43]; **(12)** Baltic amber, Lutetian – *Whalfera wiszniewskii*[39]; **(13)** Daohugou, Jiulongshan Fm., China, Bathonian – *Clavifemora rotundata*[31]; **(14)** Karatau, Kazakhstan, Oxfordian to Kimmeridgian – *Longipronotum benmaddoxi*, *Karataumantispa carnaria*, *Ovalofemora abbottae, O. monstruosa*, and Mesomantispidae indet.[18,21,22]; **(15)** Baissa, Siberia, Berriasian to Hauterivian – *Mesomantispa sibirica*[28,29]; **(16)** Yixian Fm., Barremian – *Archaeodrepanicus acutus*, *A. nuddsi*, *Archaeodrepanicus* sp., and *Sinomesomantispa microdentata*[31]; **(17)** Messel, Germany, Ypresian/Lutetian – *Symphrasites eocenicus*[29]; **(18)** Bembridge marls, UK, Priabonian – *Vectispa relicta*[10,35,36]; **(19)** Céreste, France, middle Oligocene – *Prosagittalata oligocenica*[37]; **(20)** Dauphin, France, middle Oligocene – *Climaciella*? *henrotayi*[37]; **(21)** Mexican amber, Burdigalian to Serravallian – *Dicromantispa electromexicana*[24]; **(22)** Dominican amber, Burdigalian – *Dicromantispa moronei* and *Feroseta prisca*[23,24]; **(23)** Dobbertin, Germany, early Toarcian – *Liassochrysa stigmatica*[33]; **(24)** Karatau – *Promantispa similis*[32]; **(25)** San Just amber, Spain, middle to upper Albian – *Aragomantispa lacerata* gen. et sp. nov. (this paper); **(26)** Kzyl-Zhar, Kazakhstan, Turonian – *Gerstaeckerella asiatica*[34]; **(27)** Burmese amber – *Doratomantispa burmanica* (unknown subfamilial relationships)[25].

B. Taxa from which depicted types of raptorial foreleg's integumentary specialisations present on the forefemora (A–L), foretibiae and/or foretarsi (i–viii) have been extracted. Types of setae have been grouped by size and the proportion between the length of the integumentary process and that of the modified seta. Structures drawn from photographs taken from the literature or from specimens examined in this work. This is not an exhaustive list, i.e., a given structure can be present in taxa not mentioned here. Grey areas represent the basal part of modified setae inserted within the integumentary process and visible by transparency. Forefemoral integumentary specialisations: **A** – *Mantispidiptera enigmatica*[42]; **B left** – *Gerstaeckerella gigantea*, *Rhachibermissa phenax*, *R. splendida*, and *Theristria delicatula*[42,55] (this paper), **B right** – *Paraberotha acra*[16,41]; **C left** – *Chimerhachiberotha acrasarii*, *R. phenax*, *R. splendida*, *Spinoberotha mickaelacrai*, *Theristria delicatula* and *A. lacerata*[16,42] (this paper), **C middle** – *Paraberotha acra*[16,42], **C right** – *Creagroparaberotha groehni*[19]; **D left** – *A. lacerata* and likely *Micromantispa cristata*[26] (this paper), **D middle left** – *G. gigantea* and *T. delicatula*[55] (this paper), **D middle right** – *Rhachiberotha signifera*[14], **D right** – *Micromantispa cristata*[26]; **E left** – *C. groehni*[19], **E right** – *R. signifera*[14]; **F** – *M. cristata*[26]; **G left** – *A. lacerata* (this paper), **G middle** – *Doratomantispa burmanica*[25], **G right** – *A. lacerata* (this paper); **H left** – *Archaeodrepanicus nuddsi*[31], **H middle** – *Ovalofemora abbottae*[22], **H right** – *Clavifemora rotundata*[31]; **I** – *Anchieta notha*, *Trichoscelia varia*[14] (this paper); **J left** – *A. notha* (this paper), **J right** – *Mantispa styriaca*[60]; **K left** – *G. gigantea* and *T. delicatula*[55] (this paper), **K right** – *Mantispa tenella*[60]; **L** – *G. gigantea*, *T. delicatula*, and *Mantispa styriaca*[55,60] (this paper). Foretibial and/or foretarsal integumentary specialisations: **i** – *P. acra* and likely *Raptorapax terribilissima*[16,41,44]; **ii** – *Karataumantispa carnaria*[18,22]; **iii** – *Clavifemora rotundata*[31]; **iv** – *A. lacerata*, *A. notha*, *Gerstaeckerella gigantea*, *T. delicatula*, and *T. varia*[14,55] (this paper); **v** – *Albertoberotha leuckorum*, *C. groehni*, *M. cristata*, *P. acra*, and *R. splendida*[16,19,26,41,42,46]; **vi** – *A. leuckorum* and *C. groehni*[19,46]; **vii** – *A. lacerata* (this paper); **viii** – *T. delicatula* (this paper).

All references are the same than those used in the main text.
